# Efficacy and safety of uncovered self‐expandable metal stents for distal malignant biliary obstruction in unresectable non‐pancreatic cancer

**DOI:** 10.1002/deo2.383

**Published:** 2024-05-31

**Authors:** Fumitaka Niiya, Naoki Tamai, Masataka Yamawaki, Jun Noda, Tetsushi Azami, Yuichi Takano, Fumiya Nishimoto, Masatsugu Nagahama

**Affiliations:** ^1^ Department of Internal Medicine Division of Gastroenterology Showa University Fujigaoka Hospital Kanagawa Japan

**Keywords:** biliary, distal, obstruction, safety, self‐expandable metallic stent

## Abstract

**Objectives:**

The efficacy of uncovered self‐expandable metal stents (UCSEMS) versus fully covered self‐expandable metal stents for distal malignant biliary obstruction remains controversial. Additionally, the heterogeneity of the disease conditions has been indicated in previous studies because pancreatic and non‐pancreatic cancers have different characteristics in clinical course. Therefore, the etiology of biliary obstruction necessitates investigations stratified by primary disease. This study aimed to evaluate the outcomes of UCSEMS, specifically for non‐pancreatic cancer‐induced distal malignant biliary obstruction.

**Methods:**

We conducted a single‐center retrospective review to evaluate the time to recurrent biliary obstruction and frequency of adverse events (AEs) in patients receiving UCSEMS for unresectable non‐pancreatic cancer‐induced malignant biliary obstruction.

**Results:**

Overall, 32 patients were enrolled in the study between January 2016 and December 2023. The median time to recurrent biliary obstruction was 140 days. AE rates were low at 3.1% for both pancreatitis and cholecystitis, suggesting a potential benefit of UCSEMS in reducing post‐procedural AEs.

**Conclusion:**

UCSEMS may reduce the risk of post‐procedural AEs and should be considered in patients at high risk of post‐endoscopic retrograde cholangiopancreatography pancreatitis. However, the patency period may be shorter, necessitating future comparative research with fully covered self‐expandable metal stents to determine the optimal stent choice.

## INTRODUCTION

Distal malignant biliary obstruction (dMBO) is a common condition requiring immediate treatment. Endoscopic retrograde cholangiopancreatography (ERCP) with biliary stent placement is considered the first‐line intervention for relieving obstruction.^1^ Various studies have been conducted on the choice of biliary stents, and several meta‐analyses have shown that self‐expandable metal stents (SEMS) have longer patency and fewer adverse events than plastic stents (PS).^2^, ^3^ Based on these findings, the guidelines of the European Society of Gastrointestinal Endoscopy recommend SEMS placement as the primary option.^4^ The three types of SEMS include uncovered SEMS (UCSEMS), fully covered SEMS (FCSEMS), and partially covered SEMS. However, the most beneficial stent type for dMBO remains unclear.

The most common adverse event (AE) following UCSEMS placement is tumor ingrowth‐induced occlusion (16%–46%), usually leading to cholangitis and recurrent jaundice.^5^ Although FCSEMS can reduce this complication, stent occlusion can still occur due to the formation of adherent bacterial biofilms, sludge, overgrowth, and migration. Other AEs, such as cholecystitis and pancreatitis, have also been reported.

However, no consensus exists on whether FCSEMS or UCSEMS performs better regarding stent patency, and various studies are ongoing. The inconsistency in conclusions about the efficacy of UCSEMS and FCSEMS in the literature can be attributed to several factors, one of which is the heterogeneity of the disease conditions. Most reports predominantly include pancreatic cancer cases; however, the pathophysiology of pancreatic and non‐pancreatic cancers evidently differs. Additionally, non‐pancreatic cancers have a higher risk of post‐ERCP pancreatitis (PEP) than pancreatic cancers,^6^ suggesting the need to consider these disease conditions separately.

At our institution, considering the risks of pancreatitis and cholecystitis, UCSEMS has been the treatment of choice for non‐pancreatic cancer‐induced dMBO because they do not obstruct the pancreatic orifice. However, only a few reports exist on the outcomes of UCSEMS, specifically for non‐pancreatic cancer‐induced dMBO. Therefore, this study aimed to evaluate the outcomes of UCSEMS, specifically for non‐pancreatic cancer‐induced dMBO.

## METHODS

### Study design and patients

This was a single‐center, retrospective analysis focusing on cases in which UCSEMS were used to manage unresectable non‐pancreatic cancer‐induced dMBO from January 2016 to December 2023.

The inclusion criterion was UCSEMS placement for dMBO not attributed to pancreatic cancer, whereas the exclusion criteria were as follows: (1) surgically altered gastrointestinal anatomy, except those with B‐I reconstruction, (2) suspected ampullary tumors, (3) instances of duodenal stenosis, (4) a hilar stricture (not ≥10 mm below the hilar bifurcation), and (5) concurrent pancreatitis.

Ethical compliance was ensured in accordance with the guidelines set by the Institutional Review Board of the hospital, and the study was conducted in accordance with the principles of the Declaration of Helsinki.

### ERCP procedure

All patients in this study underwent ERCP under general anesthesia. The procedures were performed using JF260V, TJF260V, or TJFQ290V (Olympus Medical Systems Corporation) duodenoscopes, and the SEMSs were conventionally inserted endoscopically. This process involved obtaining a cholangiogram to evaluate the biliary stricture, followed by guidewire insertion. Subsequently, the delivery system was navigated into the bile duct over the guidewire to facilitate stent placement. The decision to perform an endoscopic sphincterotomy was made at the attending endoscopist's discretion. An uncovered ZEOSTENT (ZEON Medical Inc.) was used in all cases.

### Treatment for AEs post‐UCSEMS placement

Balloon cleaning is recommended in cases of sludge‐induced recurrent biliary obstruction (RBO). For obstructions due to ingrowth or overgrowth, an additional stent should be placed. However, if migration is the issue, the stent should be removed and repositioned. For pancreatitis, conservative management should be initially attempted, and if no improvement occurs, a pancreatic duct stent should be placed. Conservative treatment is the first line of action in cholecystitis cases. If no improvement occurs, percutaneous transhepatic gallbladder aspiration or drainage should be performed. However, endoscopic ultrasound‐guided gallbladder drainage should be considered if these measures fail to improve the condition.

### Outcomes and definitions

The time to RBO (TRBO) was this study's primary outcome, while the secondary outcomes included the rate of RBO, incidence of AEs, and severity of AEs. AEs and their severity were defined according to the Tokyo Criteria 2014.^7^ Based on these criteria, RBO is characterized by the occlusion or symptomatic migration of the stent. TRBO was defined as the duration from stent placement to stent dysfunction or death, whichever occurred first. Stent dysfunction was defined as a stent occlusion or symptomatic migration. Reintervention was performed in cases where stent occlusion was suspected. The severity of AEs was graded according to the lexicon guidelines of the American Society of Gastrointestinal Endoscopy.^8^ Technical success was defined as the accurate deployment of the SEMS at the target location. In contrast, functional success was defined as achieving a 50% reduction or normalization of serum bilirubin levels within 14 days of SEMS placement. Tumor involvement at the cystic duct orifice was determined based on evidence of tumor extension around the occlusive cholangiopathy, as revealed by various imaging modalities. These modalities include cholangiography, computed tomography, endoscopic ultrasonography, and magnetic resonance imaging.

### Statistical analysis

Continuous data are expressed using median values with ranges and were compared using the Mann–Whitney U test. Categorical variables are presented as proportions and were compared using Fisher's exact test. TRBO was estimated using the Kaplan–Meier method and compared across different groups as follows: groups with or without intraductal placement of SEMS, those who did or did not receive chemotherapy post‐SEMS placement, and those with bile duct cancer and other cancer types. Comparisons were performed using the log‐rank test, and statistical significance was set at *p* < 0.05. All statistical analyses were performed using R version 3.4.1 (The R Foundation for Statistical Computing).

## RESULTS

### Patient characteristics

Table 1 presents the patient characteristics. A total of 32 patients who underwent treatment with UCSEMS were enrolled between January 2016 and December 2023. The median age was 79 (range: 59–90) years. Among these patients, the majority were male, accounting for 65.6% (21/32). The primary disease was bile duct cancer in 56.3% (18/32) of cases. Other primary diseases included gallbladder cancer in 3.1% (1/32) and various other cancer types in 43.8% (14/32). Additionally, instances of pancreatic duct dilation, the status post‐cholecystectomy, and the involvement of the tumor at the orifice of the cystic duct were 0, 6.3% (2/32), and 34.4% (11/32), respectively. The rate of cholangitis pre‐stent placement was 53.1% (17/32), and the administration of chemotherapy post‐stent placement was 31.2% (10/32), with two cases showing partial response, two cases showing stable disease, and six cases showing progressive disease.

**TABLE 1:** Patient characteristics.

	UCSEMS
	*n* = 32
Age, median (range), years	79 (59–90)
Sex, male, *n* (%)	21 (65.6)
Primary disease, *n* (%)	
Bile duct cancer	18 (56.3)
Gallbladder cancer	1 (3.1)
Other cancers	14 (43.8)
Hepatocellular carcinoma	2 (6.3)
Gastric cancer	1 (3.1)
Malignant Lymphoma	1 (3.1)
Lymph node metastasis	9 (28.1)
Pancreatic duct dilation, *n* (%)	0
Post‐cholecystectomy, *n* (%)	2 (6.3)
Tumor involvement at the orifice of the cystic duct, *n* (%)	11 (34.4)
Cholangitis before stent placement, *n* (%)	17 (53.1)
Chemotherapy after stent placement, *n* (%)	10 (31.2)
Complete response	0
Partial response	2
Stable disease	2
Progressive disease	6

Abbreviation: UCSEMS, uncovered self‐expandable metallic stent.

### Endoscopic procedural outcomes

Table 2 summarizes the endoscopic procedures used in this study.

**TABLE 2:** Endoscopic procedure.

		UCSEMS
		*n* = 32
Plastic stent before inclusion, *n* (%)		30 (75)
Papillary intervention, *n* (%)		32 (100)
EST, *n* (%)	29 (90.6)	
EPBD, *n* (%)	3 (9.3)	
Diameter of the stent, *n* (%)	8/10 mm	6 (18.8)/26 (81.3)
Length of the stent, *n* (%)	60/80 mm	17 (53.1)/15 (46.9)
Intraductal placement of SEMS, *n* (%)		14 (43.8)
Pancreatic duct stent placement to prevent PEP, *n* (%)		1 (3.1)
Prophylactic rectal NSAIDs use, *n* (%)		0

Abbreviations: EPBD, endoscopic papillary balloon dilation; EST, endoscopic sphincterotomy; NSAID, non‐steroidal anti‐inflammatory drug; PEP, post‐endoscopic retrograde cholangiopancreatography pancreatitis; UCSEMS, uncovered self‐expandable metal stents.

A plastic stent was placed before inclusion in 75% (30/32). Endoscopic sphincterotomy and endoscopic papillary balloon dilation were performed beforehand in 90.6% (29/32) and 9.3% (3/32). A 10‐mm diameter and 60‐mm length stent was used in 81.3% (26/32) and 53.1% (17/32), respectively. In 43.8% (14/32) of the procedures, a SEMS was deployed as an intraductal placement. To prevent PEP, a pancreatic duct stent was placed in one patient, and rectal non‐steroidal anti‐inflammatory drugs (NSAIDs) were not used in any of the cases.

### Outcomes of UCSEMS placement for non‐pancreatic cancer‐induced dMBO

**FIGURE 1:**
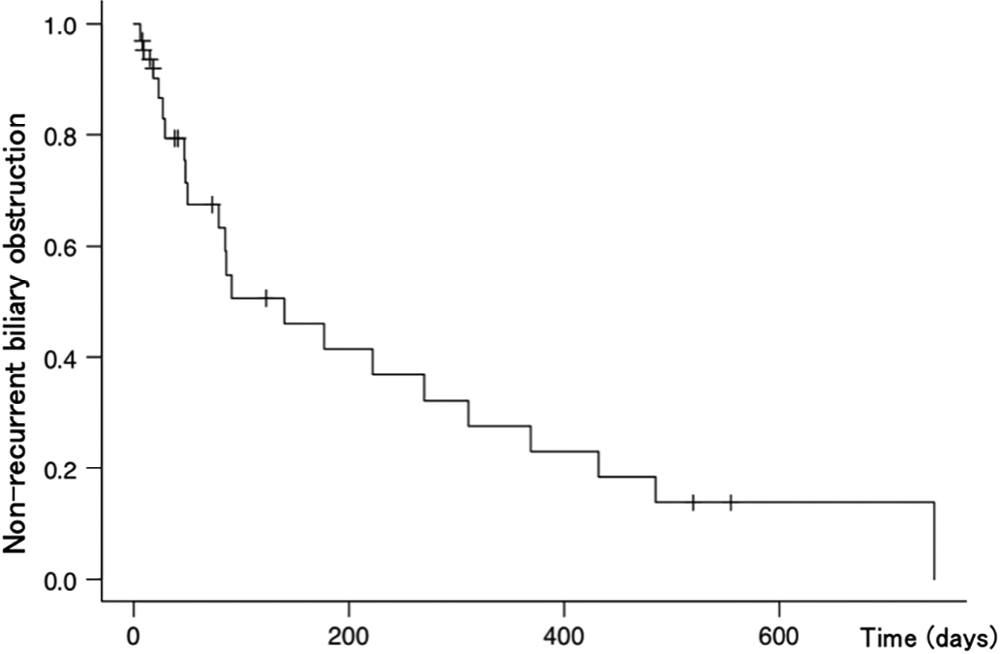
**Kaplan–Meier curve showing the time to recurrent biliary obstruction**. Kaplan–Meier analysis shows a median time to recurrent biliary obstruction of 140 days.

Table 3 shows the outcomes of the UCSEMS placement. The technical and clinical success rates were 100% (32/32). The median TRBO was 140 days (Figure 1), and the rate of RBO was 35% (14/32). The causes of RBO were diverse, with ingrowth being the most common in 25% (8/32) of the cases. Sludge was observed in 18.8% (6/32) of the cases. Overgrowth and migration were not reported as a cause of RBO. AEs other than RBO were observed in 6.3% (2/32) of the cases. These AEs were categorized based on their onset, with early AEs (within 30 days of the procedure) including one case each of pancreatitis and cholecystitis, both representing 3.1% (1/32) of the patient cohort. Notably, no late AEs (after 30 days of the procedure) were reported in this group.

**TABLE 3:** Outcomes of uncovered self‐expandable metal stent placement for distal biliary obstruction due to non‐pancreatic cancer.

	UCSEMS
	*n* = 32
Technical success, *n* (%)	32 (100)
Clinical success, *n* (%)	32 (100)
Median time to RBO (range), days	140 (6–744)
RBO, *n* (%)	14 (35)
Causes of RBO, *n* (%)	
Ingrowth	8 (25)
Overgrowth	0
Sludge	6 (18.8)
Migration	0
Adverse events other than RBO, *n* (%)	2 (6.3)
Early (≤30 days)	2 (6.3)
Pancreatitis	1 (3.1)
Cholecystitis	1 (3.1)
Cholangitis	0
Late (>30 days)	0

Abbreviations: RBO, recurrent biliary obstruction; UCSEMS, uncovered self‐expandable metal stents.

**FIGURE 2:**
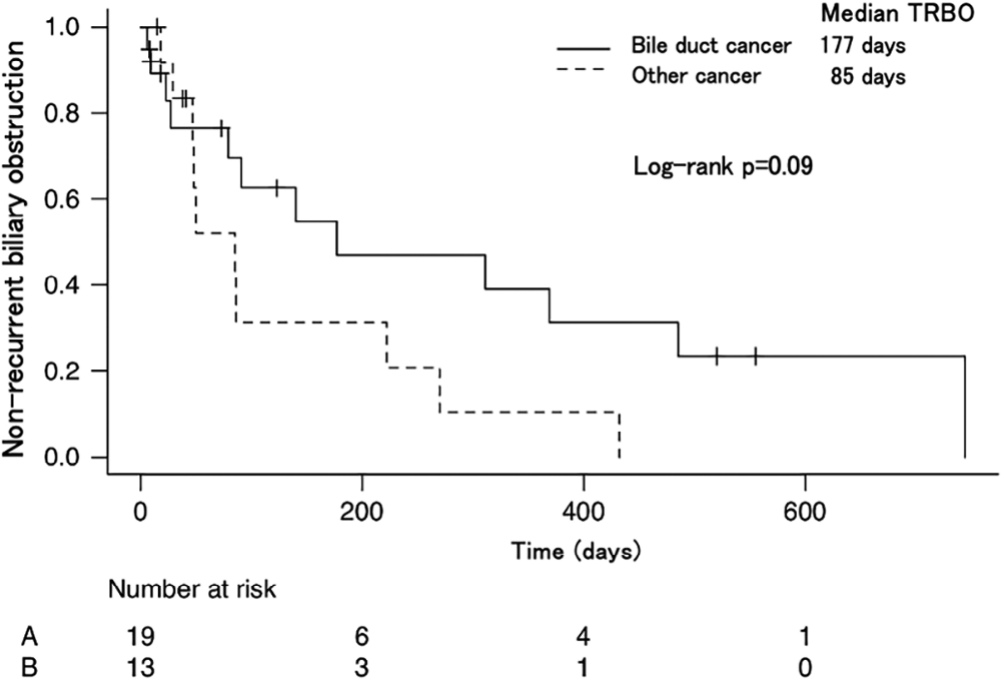
**Comparison of the median time to recurrent biliary obstruction based on the type of cancer**. The median time to biliary obstruction is 177 and 85 days in patients with bile duct cancer and other cancer types, respectively, with a log‐rank *p*‐value of 0.09.

**FIGURE 3:**
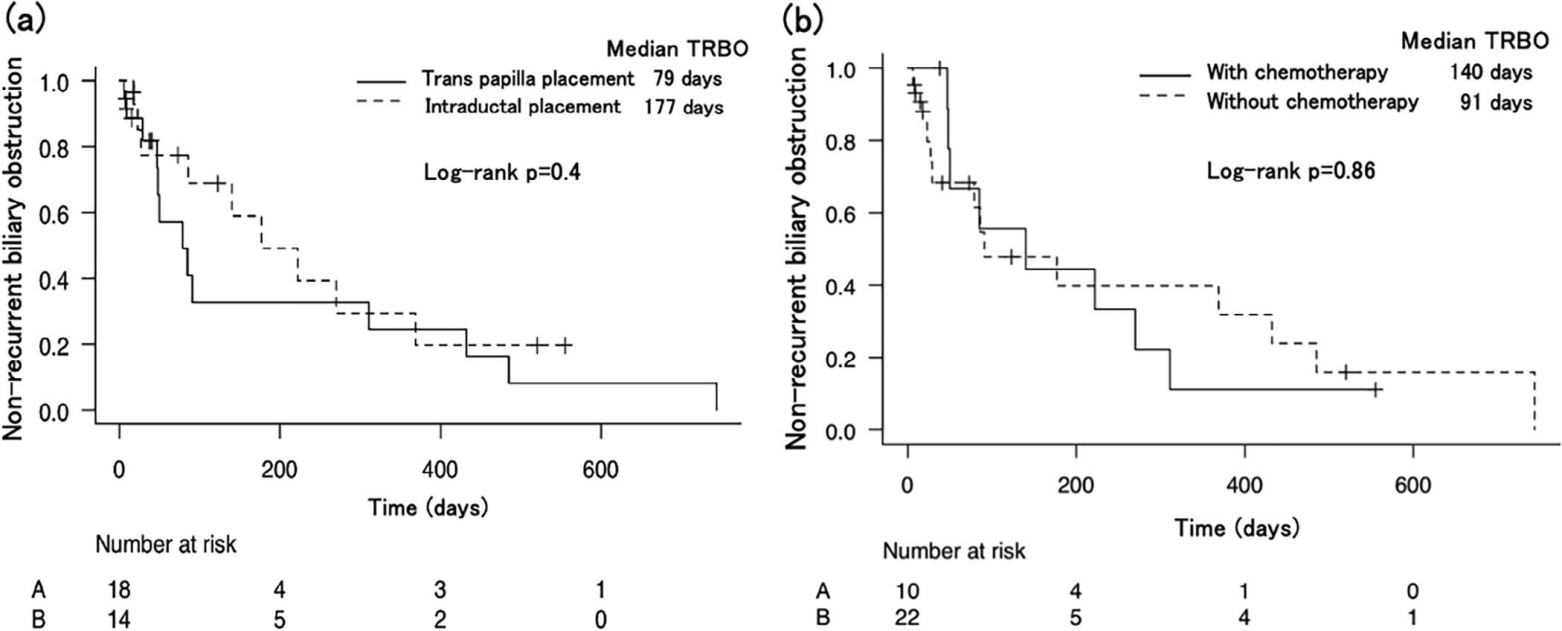
**Comparison of the median time to recurrent biliary obstruction based on SEMS placement**. (a) The log‐rank test shows that the median time to recurrent biliary obstruction is not significantly different between intraductal (177 days) and transpapillary (79 days) SEMS placements. (b) The log‐rank test shows that the median time to recurrent biliary obstruction is not significantly different between patients who received chemotherapy (140 days) and those who did not (91 days). SEMS, self‐expandable metallic stent.

Figure 2 shows the Kaplan–Meier curves of the median TRBO between cases of bile duct cancer and other cancer types. The median TRBO was comparable between the two groups, with 177 and 85 days for bile duct cancer and other cancer types, respectively (*p* = 0.09). Figure 3 displays the Kaplan–Meier curves comparing the median TRBO for cases with transpapillary and intraductal stent placement (79 vs. 177 days, *p* = 0.4) and for those with and without chemotherapy (140 vs. 91 days, *p* = 0.86).

### Characteristics of two patients who experienced AEs

Table 4 presents the characteristics of the patients with AEs. One patient had pancreatitis, and the other patient had cholangitis. The severity of the AEs was mild, and the treatment was conservative therapy in both patients. In the patient with pancreatitis, the diameter and length of the stent were 10 and 6 mm, respectively. The stent was placed across the papilla, and no prophylactic rectal NSAIDs were used. In contrast, the diameter and length of the stent were both 8 mm in the patient with cholangitis. Computer tomography performed on this patient revealed tumor involvement at the orifice of the cystic duct.

**TABLE 4:** Characteristics of the two patients who experienced adverse events (AEs).

	Age (years)/sex	AEs	Severity grade	Pancreatic duct dilation	Tumor involvement at the orifice of the cystic duct	Stent diameter (mm)	Stent length (mm)	Stent placement type	Pancreatic duct stent placement to prevent PEP	Prophylactic rectal NSAID use	Treatment
1	81/female	Pancreatitis	Mild	No	No	10	6	Across the papilla	No	No	Conservative therapy
2	81/female	Cholangitis	Mild	No	Yes	8	8	Above the papilla	No	No	Conservative therapy

Abbreviations: AEs, adverse events; NSAID, non‐steroidal anti‐inflammatory drug; PEP, post‐endoscopic retrograde cholangiopancreatography pancreatitis.

## DISCUSSION

This is the first study to focus on non‐pancreatic cancer‐induced dMBO. In this cohort, the placement of UCSEMS in patients with unresectable non‐pancreatic cancer‐related biliary obstructions demonstrated feasibility with minimal adverse events. The median TRBO was 140 days, which was shorter than that reported in previous studies.

Biliary drainage is crucial in the clinical management of patients with MBO. While achieving longer patency is undoubtedly important, it is equally vital to focus on minimizing complications, particularly to facilitate early initiation of chemotherapy. SEMSs are the recommended choice for biliary drainage in MBO. However, the selection of stents for biliary drainage, specifically UCSEMS or FCSEMS, remains controversial. Regarding patency, some prospective studies have favored FCSEMS for improved outcomes,^9^, ^10^, ^11^ while others have reported comparable results between them.^12^, ^13^, ^14^ Notably, studies have also suggested a tendency for earlier occlusion with FCSEMS than with UCSEMS.^15^ In the Japanese guidelines, FCSEMS are recommended because of their removability despite no significant difference in patency or AEs; however, this recommendation has a low evidence level.^16^

The inconsistencies in these results can be partly attributed to small sample sizes, heterogeneous disease states, and specific characteristics of the stents used.^17^ Heterogeneity in the etiology is particularly significant; most cases in previous studies predominantly involved pancreatic cancer. Therefore, differentiating pancreatic cancer from other types of cancer is crucial in such analyses. This distinction is essential because of the varying progression patterns between pancreatic and other cancer types and the higher incidence of complications, such as pancreatitis, in non‐pancreatic cancer cases.^6^ To date, studies that segregate these conditions are lacking, highlighting an imperative area for future research.

In previous reports, the occlusion rate for SEMS in dMBO was approximately 28%, with median TRBO ranging from 92 to 236 and 92 to 321 days for UCSEMS and FCSEMS, respectively.^9^, ^10^, ^11^, ^12^, ^13^, ^14^ However, the median TRBO duration was slightly shorter (140 days) in our study. Notably, ingrowth was the most common cause of occlusion, accounting for 25% of cases, whereas overgrowth and migration were not observed. Conio et al.^15^ compared FCSEMS and UCSEMS in a subgroup analysis limited to pancreatic cancer cases and found that the occlusion rate in 58 cases of UCSEMS for pancreatic cancer was 9.1% (5/58) due to ingrowth. The 25% ingrowth rate observed in our study suggests that non‐pancreatic cancers may have a higher likelihood of ingrowth than pancreatic cancers. Conversely, FCSEMS is usually associated with issues of migration and overgrowth,^18^, ^19^ indicating the need for future comparative studies between UCSEMS and FCSEMS.

SEMS placement can lead to various AEs, and managing these is crucial for maintaining quality of life in patients with dMBO. Pancreatitis and cholecystitis are two major complications associated with SEMS placement. Reports indicate that the incidence of pancreatitis and cholecystitis post‐SEMS placement ranges from 0.8% to 8.7% and 5.8% to 11.5%, respectively.^20^, ^21^, ^22^, ^23^ However, many of these reports predominantly involved cases of pancreatic cancer. Conversely, Shimizu et al.^6^ identified non‐pancreatic cancer as a risk factor for PEP, suggesting that PEP incidence in non‐pancreatic cancer cases might be higher than that previously reported. The silicone covering of the FCSEMS has also been suggested to obstruct the pancreatic or biliary orifice, leading to pancreatitis or cholecystitis. In our study, the incidences of PEP and cholecystitis were comparatively low (3.1%), indicating that UCSEMS may reduce complications post‐SEMS placement. In both patients with AEs, the severity was mild, and the AEs were treated with conservative therapy. Therefore, in patients with a high risk of pancreatitis (such as women, younger individuals, or patients with a history of pancreatitis) or those at risk of cholecystitis post‐SEMS placement due to cystic duct invasion,^24^ choosing UCSEMS over FCSEMS may be a preferable strategy. However, since an intervention to the pancreatic duct post‐SEMS placement is challenging because UCSEMS is difficult to remove, which is its disadvantage, we need to determine if PEP remained unresolved with conservative therapy. Moreover, if migration of the uncovered metal stent occurs, it may be challenging to remove it completely.

In this study, we compared TRBO based on differences in disease etiology, stent placement methods, and chemotherapy administration. However, no significant differences were observed in the TRBO across these comparisons. A tendency for a longer TRBO in biliary cancer cases was observed when comparing cases of biliary cancer with other cancer types. This could be attributed to the poor prognosis of other cancer types, which is usually due to metastatic lesions causing obstruction. Although reports suggest that intraductal stent placement may prevent the duodenobiliary reflex and achieve a longer patency period,^25^ significant differences were not found in our study in this regard. Regarding chemotherapy, the patients who received chemotherapy showed a tendency for a longer TRBO than those who did not receive chemotherapy. Kang et al.^26^ reported that patients who received chemotherapy post‐SEMS placement had better stent patency than those who did not receive chemotherapy. Therefore, based on these factors, further investigations with larger cohorts are required to validate these findings.

This study had some limitations. First, the sample size was relatively small, which may limit the generalizability of our findings. Second, the retrospective nature of this study may also introduce biases and limitations in data collection and interpretation. Third, a direct comparison with FCSEMS would have been ideal for a more comprehensive understanding of the efficacy of UCSEMS, although such comparative data were unavailable in this study. Therefore, prospective studies with larger cohorts are essential to determine the optimal stenting approach for non‐pancreatic MBO. Despite these limitations, this is the first study to focus on UCSEMS in this specific patient population of non‐pancreatic cases, which could contribute to addressing a critical issue that has previously been underexplored.

In conclusion, UCSEMS potentially lowers the complication risks in non‐pancreatic cancer cases and may be particularly beneficial for patients prone to PEP and cholecystitis. These stents may also result in a shorter TRBO. Therefore, large‐scale studies comparing UCSEMS with FCSEMS are required to determine the most effective treatment approaches for these patients.

## CONFLICT OF INTEREST STATEMENT

None.
